# Impact of Cover Crop Planting and Termination Dates on Arthropod Activity in the Following Corn

**DOI:** 10.1093/jee/toac090

**Published:** 2022-07-04

**Authors:** Gabriela Inveninato Carmona, Emily Robinson, Alexandre Tonon Rosa, Christopher A Proctor, Anthony Justin McMechan

**Affiliations:** Department of Entomology, University of Nebraska-Lincoln, NE 68583, USA; Department of Statistics, University of Nebraska-Lincoln, NE 68583, USA; Department of Agronomy, University of Wisconsin, Madison, WI 53706, USA; Department of Agronomy and Horticulture, University of Nebraska-Lincoln, NE 68583, USA; Department of Entomology, University of Nebraska-Lincoln, NE 68583, USA; Nebraska Research, Extension, and Education Center, Ithaca, NE 68033, USA

**Keywords:** cover crop, planting date, termination date, arthropod, corn

## Abstract

Relative to fallow-cash crop rotations, the addition of a cover crop can contribute to greater plant diversity and has the potential to conserve predatory arthropods. The transition of arthropods from a cover crop to a subsequent cash crop depends on several factors, such as cover crop biomass production and weather conditions. Information about the effect of cover crop planting and termination dates on arthropods in a subsequent corn system is limited. A two-year field study was conducted in Nebraska in 2018/2019 and 2019/2020 to evaluate the impact of cover crop planting and termination dates as a source for arthropods in the subsequent corn. A total of 38,074 and 50,626 arthropods were collected in the first and second year, respectively. In both years, adding a grass cover crop increased predatory arthropods but reduced yield in follow corn crop. Of the arthropods collected, Carabidae and Araneae had greater activity with cover crop biomass increments, whereas Collembola and Acari activity only increased in treatments with little or no cover crop biomass. Insect pest pressure was not significant in any treatment for either year. A cover crop planted in mid- or late-September and terminated at corn planting was identified as the best management strategy to maximize cover crop biomass, increase predator activity, and modify predator-prey dynamics. The results of this study provide growers with a cover crop management strategy to maximize cover crop biomass, beneficial arthropod activity, and potentially minimize insect pest problems; however, corn *Zea Mays* (L.) grain yield was reduced as cover crop biomass increased.

Cover crops consist of grasses, legumes, and forbs used alone or in mixtures for seasonal cover. The use of a cover crop provides environmental and agricultural benefits through erosion control, soil health, water quality improvement, and weed suppression (Bottenberg et al. 1997, CTIC 2017, Nichols et al. 2020, Menta and Remelli 2020). In addition, cover crops have been shown to change the pest and beneficial arthropods population dynamics by increasing plant diversity within the agroecosystem (Altieri et al. 1985, Smith et al. 1988, Clark et al. 1993, Carmona and Landis 1999, Shearin et al. 2008, Ward et al. 2011, Dunbar et al. 2017, Carmona et al. 2019). Due to the cover crop benefits and potential to increase ecosystem functions (Schipanski et al. 2014), cover crops have increased from 10.3 million acres in 2012 to 15.4 million acres in 2017 (USDA-NRCS 2017, CTIC 2017). Cover crop acres are expected to increase to 20 million acres in 2021 across the United States (Hamilton et al. 2017).

Several cover crop species are available for growers. Cover crop species selection depends on growers’ goals, whether it is weed suppression, nitrogen fixation, erosion reduction, or water quality. A recent systematic review of the impact of cover crop management tactics on arthropods in a cover crop-corn/soybeans systems showed that cereal rye [*Secale cereale* L. (Poaceae)] is the most studied cover crop in the USA due to its ease of establishment, high biomass production, low price, compatibility with multiple crop rotation systems, and winter hardiness (Carmona et al. 2021). Oat [*Avena sativa* L (Poaceae)] is the second most used cover crop in the USA (USDA-NRCS 2017, Wallander et al. 2021), likely as a result of its rapid biomass accumulation in the fall, reduced water consumption, less nitrogen immobilization, and its inability to survive freezing temperatures. However, because oat is a winter-killed cover crop, it might not provide as many ecosystem services compared to a winter-hardy cover crop due to its shorter period of opportunity for growth in the field (Quemada and Cabrera 1995, White et al. 2016, Kaye et al. 2019, Casey 2012). Multispecies cover crop mixes are expected to add diversity and increase biomass production (USDA 2017). Using a cereal rye and oat cover crop mix allows for the rapid emergence of oat in the fall and with the potential for greater cereal rye biomass accumulation in the spring. The combination of oat and cereal rye can potentially maximize plant biomass during the cover crop period (Chapagain et al. 2020).

Management practices can also maximize cover crop biomass by planting cover crops early in the fall or terminating late in the spring. However, wet conditions around fall harvest can delay cover crop planting. Moreover, there are no cover crop planting date guidelines to address its ideal planting period. As a result, studies are needed to determine the ideal cover crop planting period to allow growers to make informed decisions on when to plant a cover crop to maximize its benefits. Unlike the cover crop planting date, the timing of cover crop termination is based on guidelines from the Natural Resources and Conservation Service (NRCS). These guidelines consist of four zones that aim to achieve conservation benefits while minimizing the risk of yield reduction to the subsequent crop due to limited water availability (USDA-NRCS 2017). The recommendation for cover crop termination date varies from terminating a cover crop 35 d or earlier for USA states in zone 1 (western USA) to terminating before crop emergence in zone 4 (eastern USA). In general, a cereal rye cover crop should achieve at least 152 mm of extended leaf height growth before termination to increase the likelihood of agronomic benefits (USDA NRCS 2014, NRCS NE Code 340, NRCS 2007).

Previous research in cover crop agroecosystems has shown that the addition of a cover crop can increase beneficial arthropod activity and density (Laub and Luna 1992, Letourneau et al. 2011, Clark et al. 1993, Carmona and Landis 1999, Schmidt et al. 2007, Shearin et al. 2008, Davis et al. 2009), decrease pest populations (Buntin et al. 1994, Lundgren and Fergen 2010; Koch et al. 2012, 2015), and provide ecosystem services because of an increase in predation levels (Altieri et al. 1985, Lundgren and Fergen 2010). On the other hand, some studies found an increase in pest pressure when a cover crop was used (Hammond and Jeffers 1983; Hammond 1984, 1990). Carmona et al. (2019) conducted a field survey in 2017 when unexpected plant injury was observed in cornfields that were previously planted with a wheat or rye cover crop. In this survey, wheat stem maggot (*Meromyza americana* Fitch; Diptera: Chloropidae) was identified as a cover crop pest that was causing damage to the early stage corn due to late cover crop termination (Carmona et al. 2019). These studies highlight the importance of better understanding the role that cover crop management has in the risk of pests or increase in beneficial arthropods.

To date, only a few studies have documented the impact of cover crop biomass on arthropods in a cover crop-corn or soybean system (Carmona et al. 2021). Shearin et al. (2008) found that treatments with more cover crop biomass attracted a greater number of *Harpalus rufipes* Degeer during cover crop growth and retained this increased activity for a longer period compared to treatments with less biomass accumulation. In addition, Rivers et al. (2018) observed that a more complex and diverse habitat (i.e., plant residue architecture and less amount of soil exposed) supported more predatory arthropods such as Araneae, Opiliones, Staphalynidae, and Carabidae. Ward et al. (2011) found that a greater number of carabid beetles were captured in treatments with dense canopy cover compared to treatments with less canopy cover. With only a few studies conducted, the relationship between cover crop biomass production and arthropod activity is not well understood. In addition, cover crops and arthropods are likely a complex interaction between factors such as cover crop species, planting/termination date, termination method, biomass accumulation of the cover crop, and environmental conditions (Carmona et al. 2021).

Historical literature, although useful, lacks consistent measurements and the detail necessary to determine risk or benefits based on cover crop characteristics (Carmona et al. 2021). Thus, more detailed research is needed to address how management practices influence cover crop biomass in a cover crop-corn rotation and the impact its biomass has on arthropods and prey-pest-beneficial interactions. As a result, a two-year field study was conducted in eastern Nebraska to determine the impact of cover crop planting date in the fall and its termination date in the spring on arthropod activity in the subsequent corn crop. We hypothesized that management strategies that result in greater cover crop biomass would support more arthropod prey resulting in greater arthropod activity. Furthermore, we hypothesized that, if pests are present, plots with greater cover crop biomass would support more beneficial arthropods and consequently would reduce pest populations.

## Material and Methods

### Experimental Design and Field Characteristics

**Fig. 1.**
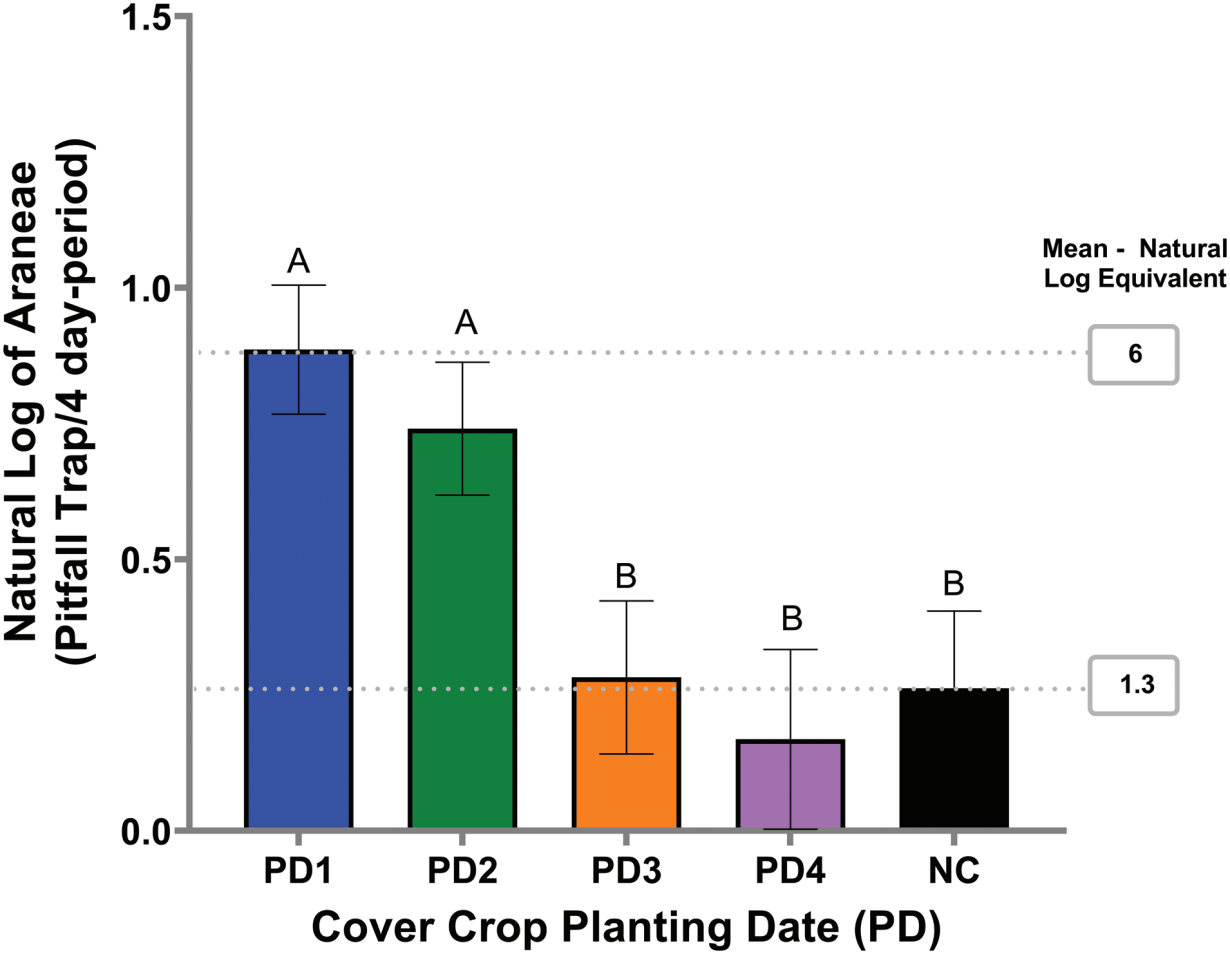
Araneae activity per cover crop planting date captured in pitfall traps in 2019. Sample error bars represent the standard error of the natural log of the means. Gray dash lines indicate the natural log – mean equivalent. PD and NC represent cover crop planting date and no-cover, respectively. Same letters represent no statistically significant difference at *P* <0.05.

Field studies were conducted over two years on separate no-till rainfed fields at the Eastern Nebraska Research, Extension, and Education Center near Mead, NE (41.168958, −96.410353 and 41.147957, −96.446446) following soybean. The experiment was conducted as a randomized complete block split-plot design (Supp Fig. 1A and B [online only]). In 2018/2019, treatments were replicated (rep) six times and four times in 2019/2020. The main plots consisted of four cover crop planting dates and a no-cover crop plot as a check with a split-plot treatment of two cover crop termination dates. Each cover crop planting date was planted based on the harvest date for a range of soybean maturity groups (0.6, 1.8, 2.5, 2.9, and 3.4) that were planted into the main plot in the spring of each year, following the methods described by Scirresi et al. (2020) for Mead, NE. Table 1 shows the cover crop planting dates for each growing season. Each main plot was 9.1 m (12 corn rows) wide by 42.6 m long. Main plots were divided into two split-plot treatments consisting of the early and late cover crop termination dates (Supp Material Fig. 1A [online only]). Split-plot treatments were 9.1 m wide and 21.3 m long. Plot size >9 m width was chosen to maintain an independent response of arthropod activity in each plot according to Prasifka et al. (2005). The herbicide glyphosate (*N*-[phosphonomethyl] glycine^3^) (2.24 kg ha^−1^) was used to terminate the cover crop. Early cover crop termination date occurred 15–18 d before planting corn, and the late cover crop termination date on the same day as planting corn (Table 1).

**Table 1.** Growing season activities (samples, measurements, and treatment application) dates for 2018/2019 and 2019/2020 cover crop-corn growing season

Activity	Cover Crop-Corn Growing Season	
	2018/2019	2019/2020
Soybean planting	8 May 2018	15 May 2019
Soybean Population (seeds ha^−1^)	303,810	345,800
Previous soybean maturity group (MG) – Cover crop planting date		
MG 0.6 – 1	12 September 2018	20 September 2019
MG 1.8 – 2	24 September 2018	27 September 2019
MG 2.5 – 3	19 October 2018	9 October 2019
MG 3.4 – 4	30 October 2018	18 October 2019
MG 2.9 – No Cover	**–**	**–**
Cover Crop Population (seeds ha^−1^)	209,950	209,950
Cover crop termination date		
Early	15 April 2019	20 April 2020
Late	29 April 2019	6 May 2020
Corn planting date	29 April 2019	5 May 2020
Corn Population (seeds ha^−1^)	79,040	79,040
Corn hybrid	P1197AM	P1244AM
Corn harvest	22 October 2019	5 October 2020
Pitfall trap sampling period		
One (before cover crop termination)	8–15 April 2019	30 April–3 May 2020
Two (at early cover crop termination)	15–19 April– 2019	21–25 April 2020
Three (at late cover crop termination)	29 April–2 May 2019	5–9 May 2020
Four (at V3 corn stage)	30 May–5 June 2019	4–9 June 2020
Five (at V6 corn stage)	10–13 June 2019	16–20 June 2020
Corn injury assessment		
V2 corn stage	30 May 2019	4 June 2020
V5-V6 corn stage	10 June 2019	16 June 2020

The cover crop consisted of a 50:50 mix by seed number of cereal rye (‘Elbon’) and oat (‘Hayden’) (Green Cover Seed Company) that were planted at a rate of 89.7 kg ha^−1^ in a 19.5 cm row spacing and 3.8 cm depth. To align with a typical production system, commercial *Bt* corn hybrid, P1197AM and P1244AM was planted across the entire study with a rate of 79,040 seeds ha^−1^ (32,000 seeds ac^−1^) in 2019 and 2020, respectively (Table 1). Both corn hybrids used in the study contained AM technology, which stands for Optimum AcreMax Insect Protection system, the details of which can be found at Difonzo (2022).

### Environmental Conditions

Temperature, average historical temperature, precipitation, and average historical precipitation data were obtained from the ‘*High Plains RCC CLIMOD, daily data for a month, MEAD 6S station’* website (High Plains 2021). Growing degree-day accumulation for each pitfall trap period was calculated by the sum of the average of the minimum and maximum temperature for each day subtracted by the minimum threshold temperature for corn (10°C) (Zur et al. 1989).

### Arthropod Sampling

Pitfall traps were used to capture ground-dwelling arthropod activity and diversity. One circular pitfall trap was placed in the center of each split-plot (Supp Fig. 1C–E [online only]) following Carmona et al. (2022). A total of five pitfall samples were taken during each growing season from each split plot. The pitfall traps were active in the field for an average of 5 d; however, due to the environmental conditions, some sampling periods were shorter or longer. Pitfall trap-specific dates per growing season are shown in Table 1. The first pitfall trap was active before any cover crop termination (hereafter: sampling period one), and second and third samples were placed in the plots at early and late cover crop termination dates (hereafter: sampling period two and three, respectively). The last two pitfall collections occurred at V3 and V6 during the early vegetative corn stages (hereafter: sampling periods four and five, respectively). The content of the pitfall traps was transferred to an individual 354 ml labeled whirl-pak bag and kept in a cold room for further analysis. All insects were counted and identified to the family level. All other arthropods collected were identified to the order level.

### Corn Injury Assessment

Corn plants were evaluated for signs of insect injury above and below ground during the V3 and V6 corn stages (sampling periods four and five, receptively) following Carmona et al. (2022). Corn damage assessments were not analyzed because of low pest pressure (>1%) across sites and years.

### Agronomic Parameters

Cover crop extended leaf height, cover crop biomass, and weed biomass were measured in each treatment just before cover crop termination following Carmona et al. (2022) protocol. Cover crop biomass reflected the treatments of this study (cover crop planting and termination dates). Total biomass (cover crop and weed biomass) was sampled from two locations in each split plot, covering a total area of 0.38 m^2^ per experimental unit. Total biomass was sampled at each pitfall trap collection period. At the end of the season, 13.7 m of the two middle rows of each split plot was harvested using a plot combine. Corn grain yield in kg ha^−1^ was calculated following:


$$\begin{array}{l} Corn\;grain\;yield\;per\;plot\;\left(corrected\right)=\frac{Plot\;grain\;weight*\left(1+\frac{13}{100}\right)}{\frac{1+plot\;grain\;moisture}{100}} \end{array}$$

$$\begin{array}{l} Corn\;grain\;yield\;\left(lbs.acre^{-1}\right)=\left(\frac{Corn\;grain\;yield\;perplot}{plot\;area}\right)/100 \end{array}$$

$$\begin{array}{l} Corn\;grain\;yield\;\left(kg.ha^{-1}\right)\;=\;Corn\;grain\;yield\;lbs.acre^{-1}*\;67.2511 \end{array}$$

### Statistical Analysis

#### Arthropod Activity

All pitfall data was standardized as a number of arthropods collected per 96-hr period to avoid bias based on the length of the sampling period. Total arthropods and arthropod families that corresponded to >1% of the total arthropods captured were analyzed using a Generalized Linear Mixed Model (PROC GLIMMIX) following a negative binomial distribution with a log function in SAS (SAS Institute, version 9.4) (SAS Institute 2008). Negative binomial distribution was chosen over the Poisson distribution after analyzing the residuals and AICCs because it allows for overdispersion and a greater frequency of zeros. For individual arthropods families, only sample periods and years with at least 30% of the pitfall traps having activity were considered for the analysis. Significant interactions with years occurred for Aphididae, Staphalynidae, Nitidulidae, Zopheridae, Anthomyiidae, Acari, and Collembola when years were included in the model (Supp Table 1 [online only]). Therefore, to simplify the understanding of the impact of cover crop planting date and termination date on arthropods, years were analyzed separated. For each year, cover crop planting date, termination date, sampling periods, and their interactions were classified as fixed effects. Random effects were rep, rep*cover crop planting date nested within rep*cover crop planting date*cover crop termination date with the scale parameter accounting for the unit level variability due to sampling periods and overdispersion of variance that often occurs in ecological data. Tukey adjustment was used on pairwise comparison tests to control for Type I error rates. Tukey LSD is reported at an *α* = 0.05 significance level. Figures were plotted with the natural log of the means and the respective untransformed means are on the right side of each graph.

#### Arthropod Community Interactions: Nonmetric Multidimensional Scaling (NMDS)

To determine if cover crop biomass impacted arthropod activity during each sampling period across all cover crop planting and termination date combinations, a nonmetric multidimensional scaling (NMDS) analysis using Bray-Curtis distances was performed, following Dunbar et al. (2016) and McDermott et al. (2021). NMDS summarizes the relationship among all variables and displays the relationships in ordination space. To determine the NMDS goodness of fit, stress values were assessed. Lower stress values indicate greater NMDS goodness of fit and therefore are desirable whereas high-stress values indicate that the ordination is arbitrary and NMDS interpretation should be avoided. To consider NMDS useful, stress values should be lower than 0.2 (Clarke 1993). The composition of each arthropod taxa and cover crop biomass becomes increasingly similar as distances between points are decreased. Cover crop biomass was added to aid in visualization and interpretation as it is strongly impacted by cover crop planting and termination date and could drive the changes in arthropod communities during each sample date and year. NMDS was performed in R (R Core Team 2018) using the *vegan* package (Oksanen et al. 2020). Functions metaMDS in R was used to compute the NMDS ordination plot that describes the relationship of the most abundant arthropod taxa with the cover crop biomass during each sampling period and year. Permutation testing (999 interactions) allowed for significant testing to describe differences in cover crop biomass on the NMDS ordinations. The ggplot2 package was then used to display the NMDS ordination plots with *k* = 3. The stress and ordination nonmetric fit *r*^2^ values were used to assess the goodness of fit between the ordination distances and the data dissimilarity. Vectors were displayed only if they had a significant relationship with the ordination.

#### Agronomic Parameters

A Linear Mixed Model (PROC GLIMMIX) was run in SAS (SAS Institute, version 9.4) with a two-way ANOVA treatment design to determine the effect of cover crop planting date, cover crop termination date, and their interaction on cover crop biomass, cover crop extended leaf height and corn grain yield. Years were analyzed separately, where for each year block variance and whole plot variance estimates were calculated with the random effect as rep nested within cover crop planting date (whole plot error), and an estimated residual variance accounted for by the assumed normal distribution of our responses. Cover crop biomass was analyzed using sample period as a repeated measure with an AR(1) correlation structure. Tukey adjustment was used on pairwise comparison tests to control for the Type I error rates. Tukey LSD is reported at an *α* = 0.05 significance level.

## Results

### Environmental Conditions

Temperature, average historical temperature, precipitation, and average historical precipitation data during each pitfall period for 2019 and 2020 are shown in Supp Figs. 2 and 3 (online only), respectively. Growing degree-day accumulation varied between pitfall sample periods and years (Supp Fig. 2A and B [online only]). For 2019, sampling periods one and two had the lowest growing degree-day accumulation, followed by sampling period five, three, and four. In 2020, the growing degree-day accumulation at sampling period three was the second lowest, and sampling period five had the second highest growing degree-day accumulation. Precipitation also varied between years (Supp Fig. 3A and B [online only]). From the first to the last pitfall sample period, a precipitation accumulation of 319 and 145 mm occurred in 2019 and 2020, respectively, where the 30-yr average accumulation precipitation is 37 mm.

### Arthropod Activity

Pitfall traps captured 38,074 and 50,626 individual arthropods in 2019 and 2020 that represented 39 and 55 different taxa, respectively. For 2019, the most dominant taxa were Acari (65.6%), Collembola (23.1%), Coleoptera: Nitidulidae (2.4%), Diptera: Anthomyiidae (2.0%), and Araneae (1.6%), composing 94.6% of the total arthropods collected. For 2020, the most dominant taxa were Collembola (44.7%), Acari (38.9%), Coleoptera: Zopheridae (3.2%), Coleoptera: Nitidulidae (2.1%), Araneae (1.9%), and Coleoptera: Staphalynidae (1.6%), composing 92.5% of the total arthropods collected.

### 2019 Growing Season

A significant planting date*termination date*sample interaction occurred for total arthropod activity (Table 2). This interaction occurred mainly as a result of the greater mean number of total arthropod activity in the planting date 1 with the early termination date (512.0) compared to no-cover crop with a late termination date (159.3) at sampling period four. In contrast, planting date 1 with an early termination did not differ from the no-cover crop with a late termination during sampling period five.

**Table 2.** *P*-values from generalized linear mixed models used to test the effect of sampling period (Sample), cover crop planting date (PD), cover crop terminating date (TD), and its interactions per year. Significant *P*-values (<0.05) are shown in bold

Year				2019							2020						
Class	Order	Family	Stats	PD*TD*Sample	PD*Sample	TD*Sample	PD*TD	Sample	PD	TD	PD*TD* Sample	PD*Sample	TD*Sample	PD*TD	Sample	PD	TD
**Insecta**	**Coleoptera**	**Staphalynidae**	***P***	N/A							0.6632	0.9734	0.5795	0.8447	**<.0001**	0.292	0.0866
			***F***								0.79	0.36	0.66	0.34	**166.72**	1.4	3.4
			**Num df, Den df**								12, 91	12, 91	3, 91	4, 14	**3, 91**	4, 12	1, 14
		**Nitidulidae**	***P***	0.7156	0.0031**	0.0902	0.7296	<.0001***	0.1108	0.4543	0.5951	0.0873	0.0033**	0.5243	<.0001	0.351	0.1817
			***F***	0.77	**2.36**	2.04	0.51	**50.76**	2.16	0.58	0.85	1.67	**4.91**	0.84	33.3	1.23	1.98
			**Num df, Den df**	16, 200	**16, 200**	4, 200	4, 25	**4, 200**	4, 20	1, 25	12, 91	12, 91	**3, 91**	4, 14	3, 91	4, 12	1, 14
		**Zopheridae**	***P***	N/A							0.3518	0.0033**	0.3117	0.1223	<.0001***	0.5154	0.5458
			***F***								1.11	**2.43**	1.21	2.2	**95.68**	0.86	0.38
			**Num df, Den df**								16, 121	**16, 121**	4, 121	4, 14	**4, 121**	4, 12	1, 14
	**Diptera**	**Anthomyiidae**	***P***	0.5555	0.3951	0.2649	0.8004	<.0001***	0.1176	0.0663	N/A						
			***F***	0.91	1.06	1.32	0.41	**55**	2.11	3.69							
			**Num df, Den df**	16, 200	16, 200	4, 200	4, 25	**4, 200**	4, 20	1, 25							
	**Hemiptera**	**Aphididae**	***P***	N/A							N/A	N/A	N/A	0.3893	N/A	0.157	0.0092***
			***F***								N/A	N/A	N/A	0.79	N/A	2.31	**9.12**
			**Num df, Den df**								N/A	N/A	N/A	1, 14	N/A	1, 11	**1, 14**
**Arachnida**	**Araneae**	–	***P***	0.5193	0.6094	0.1297	0.4444	<.0001***	0.0007**	0.5971	0.8557	0.3558	0.0082**	0.3701	<.0001***	0.0288**	0.8622
			***F***	0.94	0.87	1.8	0.96	**26.9**	**7.6**	0.26	0.63	1.11	**3.61**	1.16	**114.81**	**3.69**	0.03
			**Num df, Den df**	16, 200	16, 200	4, 200	4, 25	**4, 200**	**4, 20**	1, 25	16, 121	16, 121	**4, 121**	4, 14	**4, 121**	**4, 12**	1, 14
	**Acari**	–	***P***	0.0200**	0.0048**	0.3711	0.0401**	0.0542	0.1275	0.0542	0.9401	0.0143**	0.0006**	0.0468*	<.0001	0.2412	0.3531
			***F***	**3.21**	**4.26**	0.81	**2.95**	3.89	2.04	4.08	0.45	**2.27**	**6.29**	**3.18**	839.41	1.59	0.92
			**Num df, Den df**	**4, 50**	**4, 50**	1, 50	**4, 25**	1, 50	4, 20	1, 25	12, 91	**12, 91**	**3, 91**	**4, 14**	3, 91	4, 12	1, 14
**Collembola**	–	–	***P***	0.4449	0.821	0.5475	0.4319	<.0001***	0.0745	0.8461	0.6372	0.0626	0.5522	0.8675	<.0001***	0.0487*	0.7465
			***F***	1.01	0.62	0.72	0.99	**181.16**	2.51	0.04	0.84	1.66	0.76	0.31	**388.37**	**3.29**	0.11
			**Num df, Den df**	12, 150	12, 150	3, 150	4, 25	**3, 150**	4, 20	1, 25	16, 121	16, 121	4, 121	4, 14	**4, 121**	**4, 12**	1, 14
**Total Arthropods**			***P***	0.0182**	0.0120**	0.6551	0.281	<.0001***	0.3461	0.631	0.1217	<.0001***	0.0003**	0.1513	<.0001***	0.1435	0.1544
			***F***	**1.95**	**2.05**	0.61	1.35	**534**	1.19	0.24	1.47	**4.89**	**5.68**	2	**518**	2.1	2.27
			**Num df, Den df**	**12, 200**	**16, 200**	4, 200	4, 25	**4, 200**	4, 20	1, 25	12, 121	**16, 121**	**4, 121**	4, 14	**4, 121**	4, 12	4, 14

N/A family did not compose at least 1% of the total; therefore, arthropod taxa was not accounted for analysis. *** = <.0001 ** = <0.01 * = <0.05 significant levels.

For Acari activity, a significant planting date*termination date*sample interaction occurred (Table 2). This interaction was mainly due to lower mean number of Acari activity in the no-cover crop with a late termination date (90.6) compared to the planting date 1 with an early termination (311.9) during sampling period four. In contrast, the no-cover crop late termination date (244.5) had a greater mean number of Acari activity compared to the planting date 1 with an early termination date (102.1) at the fifth sampling period.

For Araneae, cover crop planting date was significant (Table 2), with planting dates 1 and 2 having a greater mean number of Araneae activity mean compared to planting dates 3, 4, and no-cover crop (Fig. 1).

A planting date*sample interaction was significant for Nitidulidae (Table 2). The interaction occurred mainly due to a lower mean number of Nitidulidae activity in planting date 1 (3.6) and 2 (4.1) compared to the planting date 3 (9.6), 4 (10.7), and no-cover crop (10.1) at sampling period two. In contrast, planting dates 1 (0.1) and 2 (0.1) were not different from 3 (0.1) and no-cover crop (0.1) during the other sampling periods. Anthomyiidae and Collembola were not affected by the cover crop treatments, but differences were observed between sampling periods (Supp Table 2 [online only]).

### 2020 Growing Season

Significant interactions occurred between the planting date*sample period and termination date*sample period occurred for the total arthropod activity (Table 2). The planting date*sample period interaction was a result of a greater mean number of total arthropod activity in the no-cover crop (204.4) and planting date 2 (174.2) compared to the planting date 1 (104.6) and 4 (99.5) for sampling period one. In contrast, no cover crop (26.3) had lower arthropod activity compared to the other planting dates (1: 83.9; 2: 89.2; 3: 75.9; 4: 66.7) for sampling period 3. No differences between planting dates were identified during the other sampling periods. The termination date*sample interaction occurred due to a lower mean number of total arthropod activity in the early termination date (48.4) compared to the late termination date (81.5) during sampling period three, whereas no differences were identified during any of the other sampling periods.

For Acari activity, three two-way interactions were significant, planting date*termination date, planting date*sample period, and termination date*sample period (Table 2). The planting date*termination date interaction was a result of a greater mean number of Acari activity in the early cover crop termination date (43.4) compared to the late cover crop termination date (19.9) in the planting date 1 whereas no differences occurred between termination dates in the other planting dates. The planting date*sample period interaction occurred as a result of planting date 1 (58.0) and 2 (74.4) had lower mean number of Acari activity compared to the other planting dates (*F* = 4.47; df = 4, 91; *P* = 0.0024) (planting date 3: 139.8; 4: 131.6; no-cover crop: 138.4) during sampling period five. In contrast, no differences between planting dates during the sampling periods two (*F* = 1.39; df = 4, 91; *P* = 0.2431), three (*F* = 1.97; df = 4, 91; *P* = 0.1050), and four (*F* = 1.14; df = 4, 91; *P* = 0.3436). The termination date*sample period was a result of greater mean number of Acari activity in the late cover crop termination date (2.0) compared to the early cover crop termination date (4.4) during sampling period two (*F* = 4.87; df = 1, 91; *P* = 0.0298). In contrast, the early termination date (117.9) had greater mean number of Acari activity compared to the late termination date (87.4) during sampling period five (*F* = 4.50; df = 1, 91; *P* = 0.0366).

**Fig. 2.**
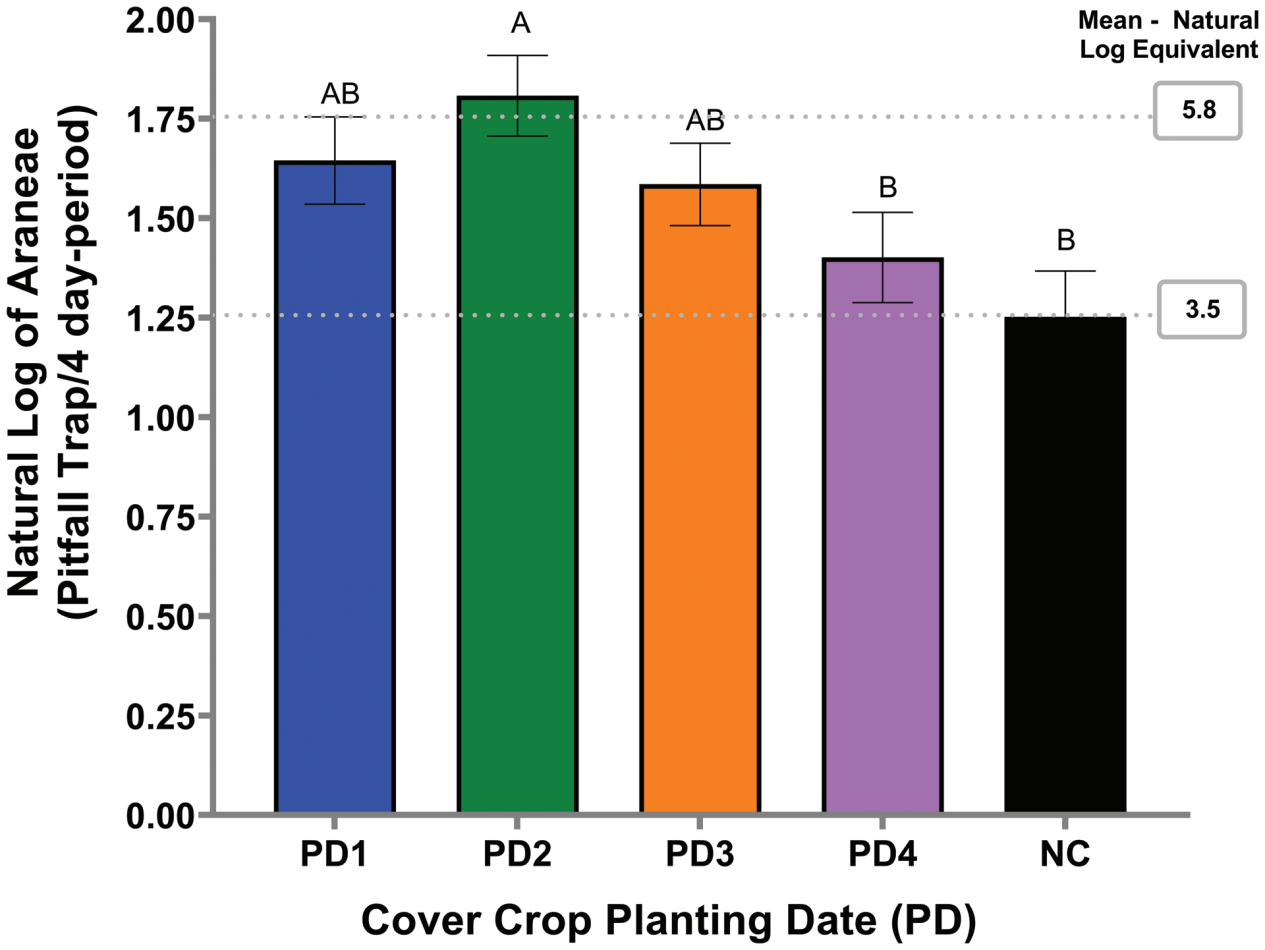
Araneae activity in natural log per cover crop termination date and per cover crop planting date from pitfall traps in 2020. Error bars indicate the standard error of the natural log of the means. PD and NC represent cover crop planting date and no-cover, respectively. Gray dash lines indicate the natural log–mean equivalent.

For Araneae, the interaction between the termination date*sample period and the main effect planting date were significant (Table 2, Fig. 2). The termination date*sample period interaction occurred due to a lower mean number of Araneae activity in the late cover crop termination date (1.4) compared to the early cover crop termination date (2.2) during sampling period three (*F* = 1.59; df = 1, 121; *P* = 0.0451) In contrast, greater mean number of Araneae activity occurred in the late cover crop termination date (22.0) compared to the early cover crop termination date (14.2) during sampling period four (*F* =10.83; df = 1, 121; *P* = 0.0013). In addition, no differences occurred between termination dates for the other sampling periods (Table 2). For the planting date main effect, planting date 2 had a greater mean number of Araneae activity compared to planting date 4 and no-cover crop but planting date 2 was not statistically different from planting date 1, and 3.

**Fig. 3.**
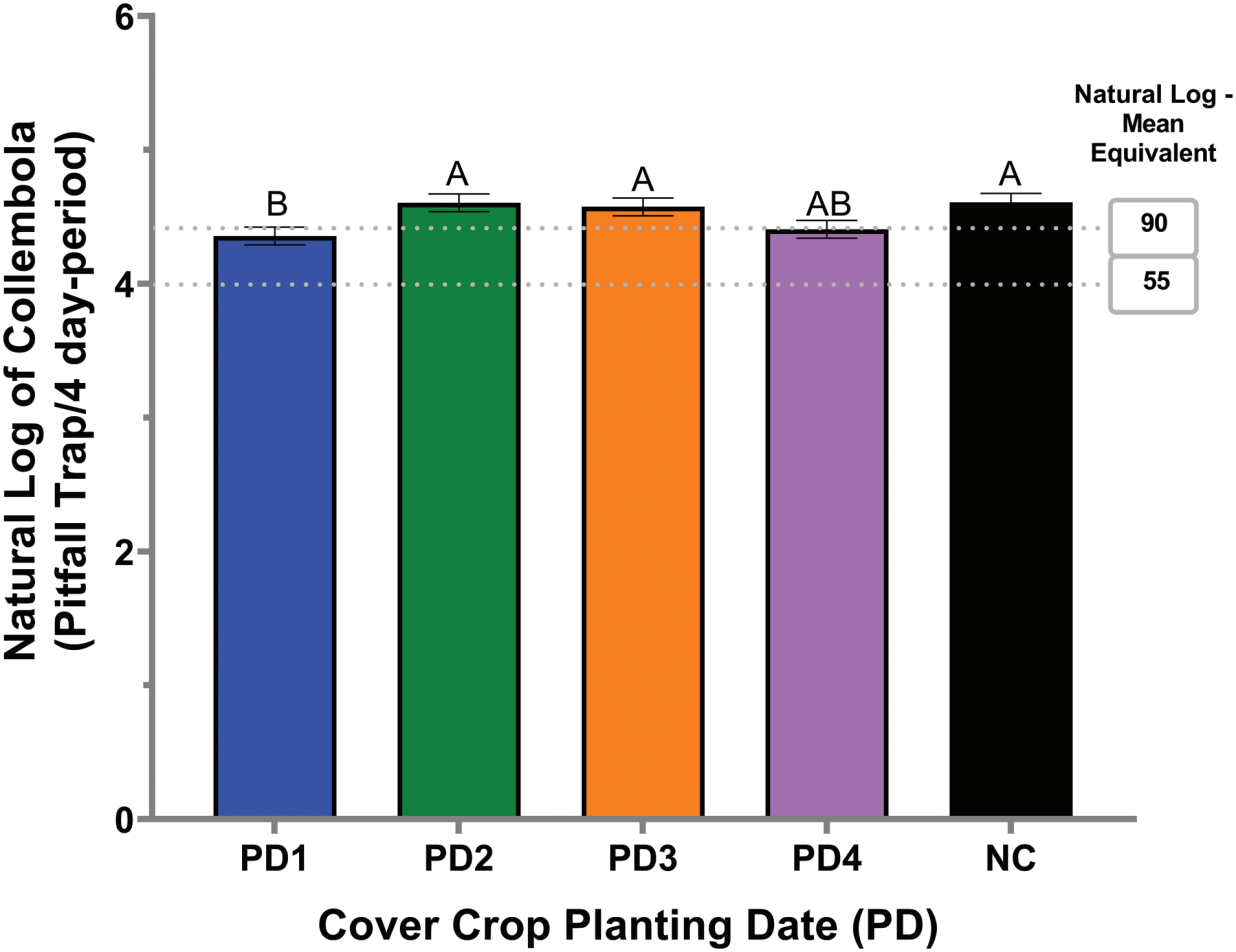
Collembola activity in natural log per cover crop planting date from pitfall traps in 2020. Error bars indicate the standard error of the natural log of the means. Gray dash lines indicate the natural log–mean equivalent. PD and NC represent cover crop planting date and no-cover, respectively. Same letters represent no statistically significant difference at *P* <0.05.

For Collembola, a planting date*sample period interaction was approaching significant, so it was further explored since the numerator degrees of freedom was greater than one (Table 2). This interaction was a result of a greater mean number of Collembola activity in the no-cover crop (175.9) and planting date 2 (148.4) compared to planting date 1 (81.5) and 4 (74.5) during sampling period one (*F* = 6.83; df = 4, 121; *P* <.0001). In contrast, only the no-cover crop (54.6) supported a greater mean number of Collembola activity compared to planting date 1 (34.1) and planting date 4 (35.9) at sampling period two (*F* = 3.84; df = 1, 121; *P* = 0.02148) and no differences between planting dates were identified during the other sampling periods (Table 2). However, a significant cover crop planting date was also identified (Table 2). The cover crop planting date 1 had a lower mean number of Collembola activity compared to planting dates 2, 3, and the no-cover crop treatment but was not different from planting date 4 (Fig. 3).

For Coleopterans, a termination date*sample period interaction was significant for Nitidulidae and a planting date*sample period was significant for Zopheridae (Table 2). The termination date*sample period interaction for Nitidulidae occurred because of a lack of differences between termination dates during sampling periods one (*F* = 0.47; df = 1, 91; *P* = 0.4931) and two (*F* = 2.15; df = 1, 91; *P* = 0.1456). In contrast, a greater mean number of Nitidulidae activity was collected in the late termination date (18.2) compared to the early termination date (11.6) during sampling period four (*F* = 9.61; df = 1, 91; *P* = 0.0026). Similar but less significant differences occurred at sampling period five (*F* = 4.25; df = 1, 91; *P* = 0.0420). For Zopheridae, the planting date*sample period interaction was a result of planting date 1 having a lower mean number of Zopheridae activity when compared to planting date 4 during sampling periods one, two, and three. In contrast, no difference occurred between those two planting dates during sampling period four. Greater mean number of Zopheridae activity was also identified in planting date 1 compared to planting date 4 at sampling period five.

**Fig. 4.**
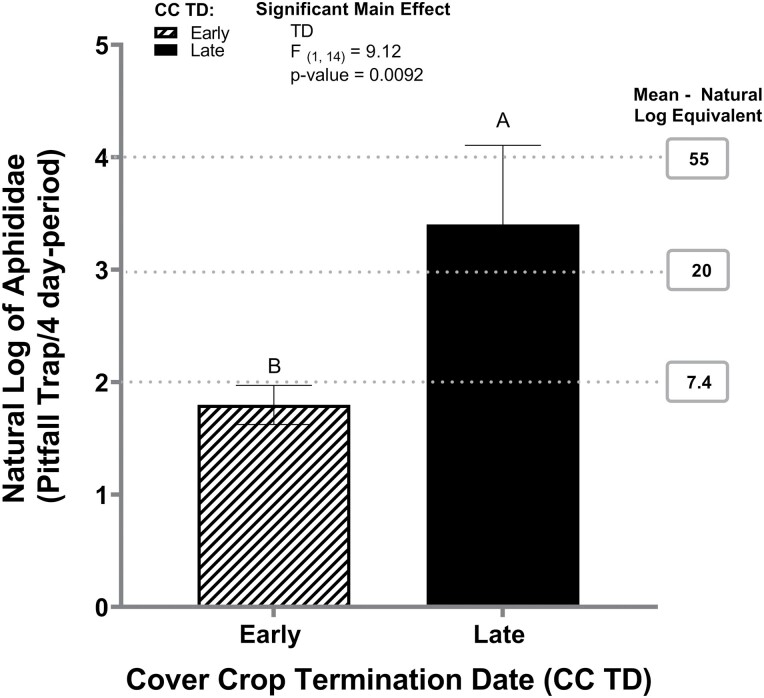
Aphididae activity in natural log per cover crop termination date from pitfall traps in 2020. Error bars indicate the standard error of the natural log of the means. Gray dash lines indicate the natural log–mean equivalent. TD represents cover crop termination dates. Same letters represent no statistically significant difference at *P* <0.05.

Anthomyiidae was impacted by the termination date, where the early cover crop termination date (1.97) had a greater mean number of Anthomyiidae activity compared to the late cover crop termination date (1.51) (Table 2). Cover crop termination date was also significant for Aphididae (Table 2), where the late cover crop termination date had a greater Aphididae activity compared to the early cover crop termination date (Fig. 4).

### Arthropod Community Interactions: NMDS

#### 2019 Growing Season

**Fig. 5.**
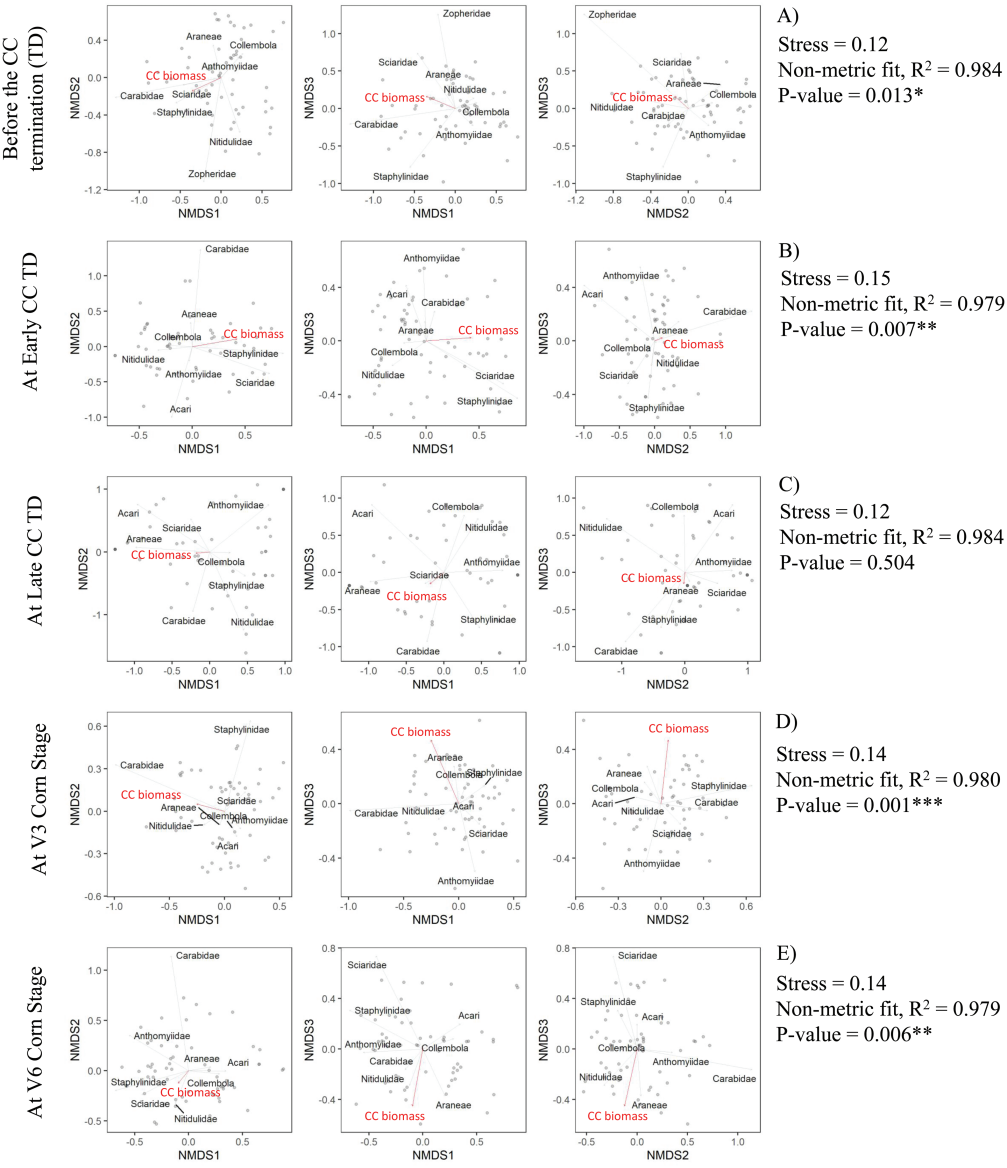
Nonmetric multidimensional scaling (NMDS) ordination results for cover crop biomass and the most abundant arthropod taxa composition as captured in pitfall traps during the before cover crop termination (A), at the early cover crop termination (B), at the late cover crop termination (C), at the V3 corn stage (D), and at the V6 corn stage (E) sampling periods in 2019.

Analysis of NMDS reached a solution with stress lower than 0.20 for all sampling periods with stress values of 0.12, 0.15, 0.12, 0.14, 0.14, for sampling periods one, two, three, four, and five, respectively (Fig. 5). Additionally, NMDS ordination distances varied with sampling periods, where ordination nonmetric fit *R*^*2*^ was 0.984, 0.979, 0.984, 0.980, and 0.979 for sampling periods one, two, three, four, and five, respectively (Fig. 5). The permutation test of correlation coefficients (999 iterations), which allows for testing the significance of the cover crop biomass on the NMDS ordinations, showed that cover crop biomass significantly impacted the arthropod activity for four of the five sampling periods. Cover crop biomass significantly impacted arthropod activity during sampling periods one (*P* = 0.0131), two (*P* = 0.0077), four (*P* = 0.0011) and five (*P* = 0.0063) (Fig. 5).

#### 2020 Growing Season

**Fig. 6.**
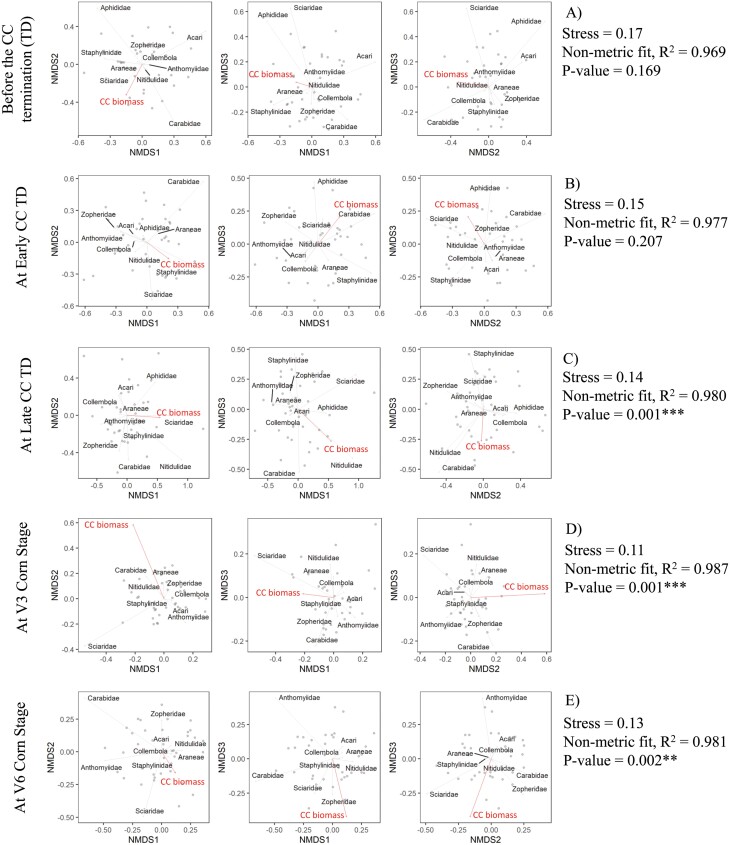
Nonmetric multidimensional scaling (NMDS) ordination results for cover crop biomass and the most abundant arthropod taxa composition as captured in pitfall traps during the before cover crop termination (A), at the early cover crop termination (B), at the late cover crop termination (C), at the V3 corn stage (D), and at the V6 corn stage (E) sampling periods in 2020.

Analysis of NMDS reached a solution with stress lower than 0.20 for sampling periods with stress values of 0.17, 0.15, 0.14, 0.11, and 0.13 for sampling periods one, two, three, four, and five, respectively (Fig. 6). Additionally, NMDS ordination distances varied with sampling periods, where ordination nonmetric fit *R*^*2*^ was 0.969, 0.977, 0.980, 0.987, and 0.981 for sampling periods one, two, three, four, and five, respectively (Fig. 6). Permutation test of correlation coefficients (999 iterations) indicated that cover crop biomass significantly impacted the arthropod activity during the 2020 season for sampling periods three (*P* = 0.0010), four (*P*= 0.0010), and five (*P* = 0.0020) (Fig. 6).

## Agronomic Parameters

### Cover Crop Dry Biomass, Extended Leaf Height, and Corn Grain Yield

Supp Table 2 (online only) shows the dry cover crop and weed biomass per sampling period. Weed species were not recorded; however, based on field observations, the most common weed species present was henbit (*Lamium amplexicaule* L.).

### 2019 Growing Season

**Fig. 7:**
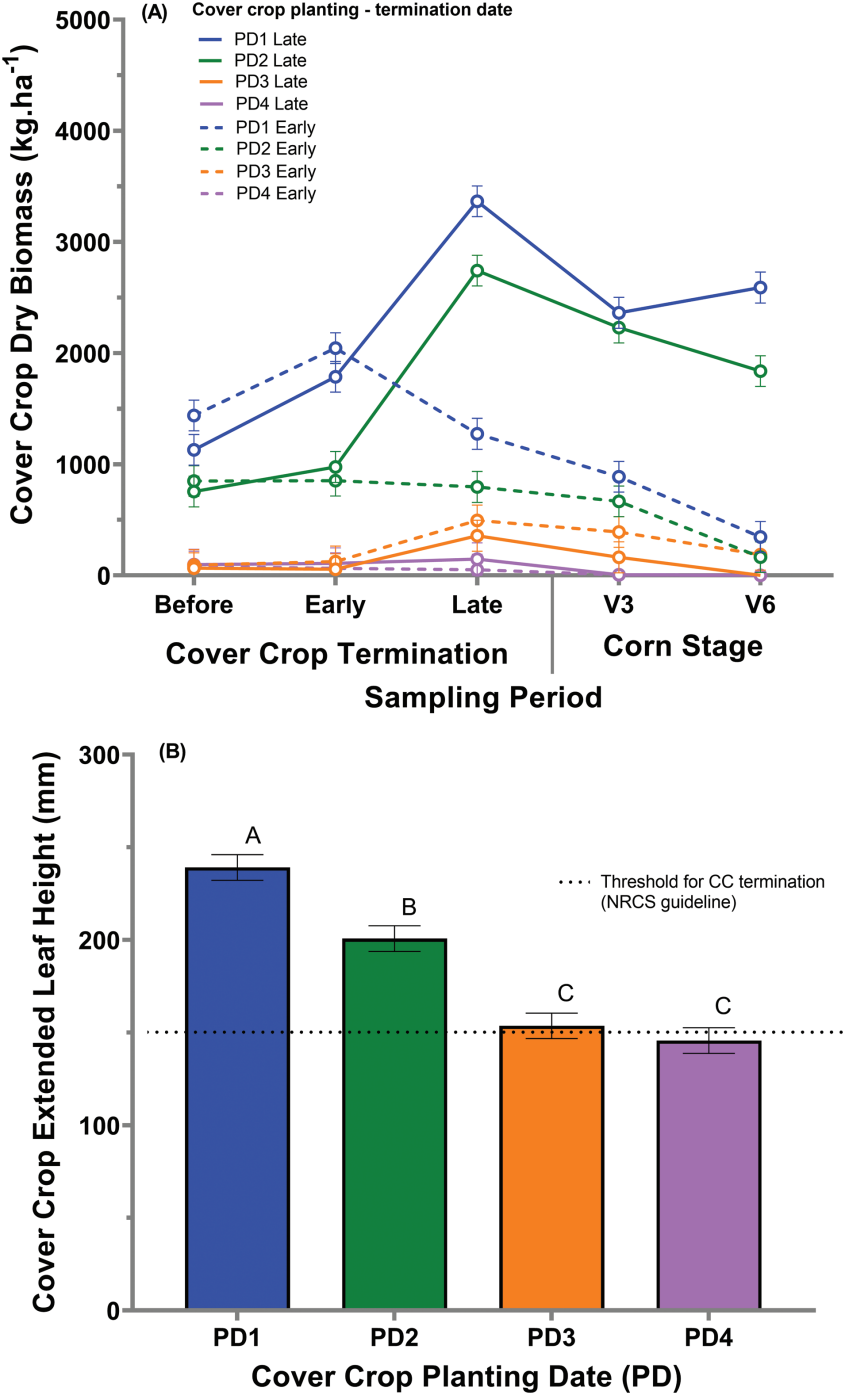
Cover crop dry biomass per cover crop planting date, cover crop termination date, and sampling period (A) and extended leaf height (ELH) per cover crop planting date (B) for 2019. PD represents the cover crop planting dates. Error bars represent the standard error of the means. The same letters indicate no statistically significant difference at *P* <0.05.

The cover crop biomass varied from 78.93 kg ha^−1^ (planting date 4 with early cover crop termination date during sampling period one) to 3365.18 kg ha^−1^ (planting date 1 with a late cover crop termination date during sampling period three). A significant three-way interaction occurred between planting date*termination date*sample period (*F* =16.66; df = 16, 199; *P* <.0001) (Fig. 7A). For planting date 1, there was no difference between early and late termination dates during sampling period two (*F* =2.13; df = 1, 199; *P =* 0.1462), while planting date 1 late termination had greater cover crop biomass compared to planting date 1 early termination during sampling period three (*F* =139.44; df = 1, 199; *P <*.0001). A similar situation occurred for planting date 2, with no differences between early and late termination dates identified during sampling period two (*F* =0.49; df = 1, 199; *P =* 0.4854). In contrast, planting date 2 with a late termination had greater cover crop biomass compared to planting date 2 early during sampling period three (*F* =99.62; df = 1, 199; *P <*.0001).

All planting and termination date treatment combinations for 2019 met the NRCS extended leaf height threshold of 152 mm at the time of the cover crop termination. The extended leaf height varied from 154 mm (planting date 4 with an early cover crop termination) to 256 mm (planting date 1 with a late cover crop termination). A cover crop planting date main effect was significant for extended leaf height (*F* = 3.94; df = 4, 12; *P =* 0.0288) (Fig. 7B). Extended leaf height decreased with a delay in cover crop planting date.

Corn grain yield varied from 8,566 kg ha^−1^ in cover crop planting date 2 to 10,549 kg ha^−1^ for the no-cover crop treatment. The cover crop planting date main effect was significant (*F =* 3.56; df = 4, 12; *P =* 0.0238). The no cover crop (10,549 kg ha^−1^) treatment grain yield was greater than cover crop planting date 2 (8,566 kg ha^−1^), but was not different from cover crop planting date 1 (9,376 kg ha^−1^), 3 (9,843 kg ha^−1^), and 4 (9,710 kg ha^−1^).

### 2020 Growing Season

**Fig. 8.**
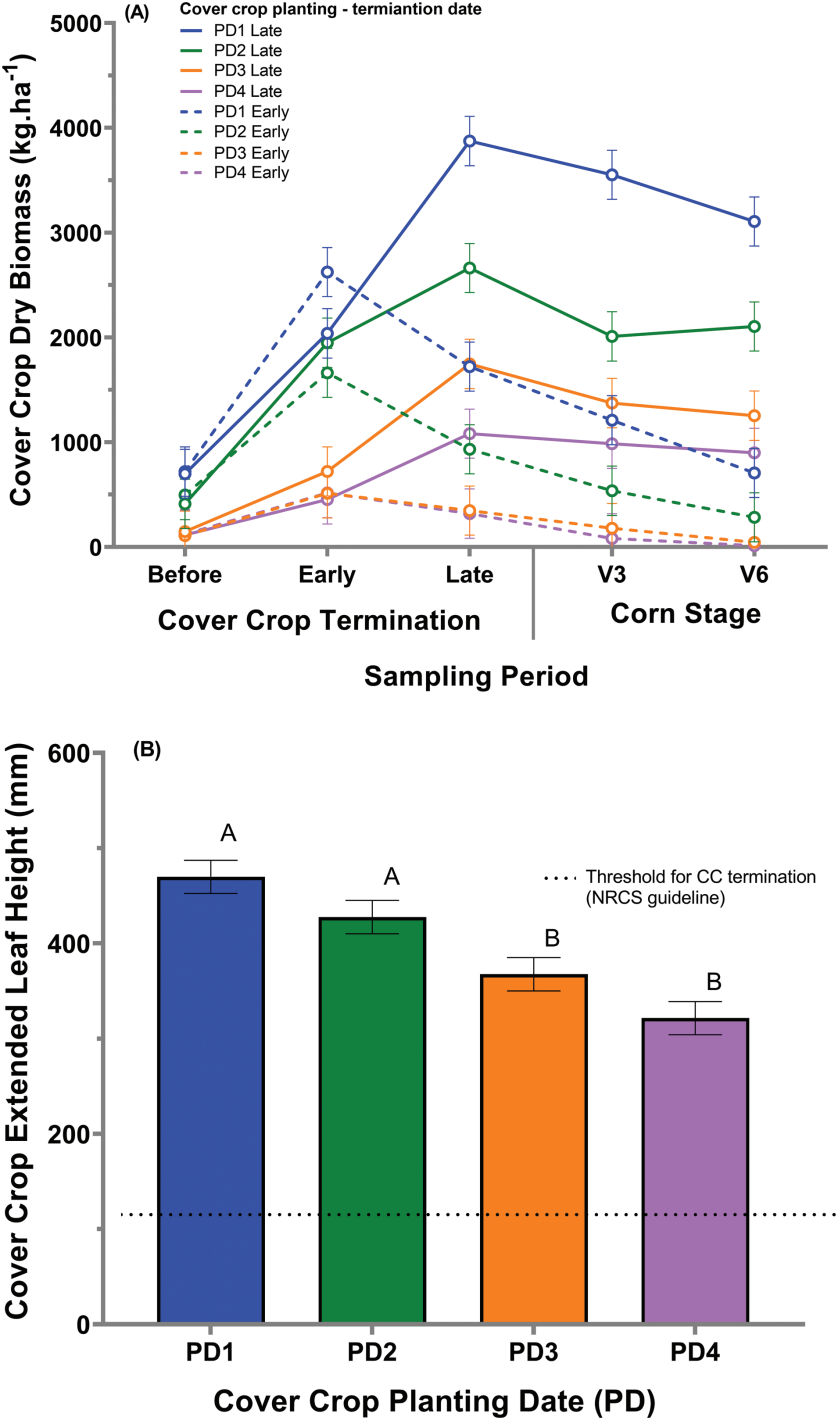
Cover crop dry biomass (A) per cover crop planting date and cover crop termination date, and extended leaf height (ELH) per cover crop planting date (B) for 2020. PD represents the cover crop planting dates. Error bars represent the standard error of the means. The same letters indicate no statistically significant difference at *P* <0.05.

The cover crop biomass varied from 121.2 kg ha^-−1^ for planting date 4 with an early cover crop termination date during sampling period one to 3873.8 kg ha^−1^ for planting date 1 with a late cover crop termination date during sampling period three. Cover crop biomass had a significant three-way interaction for planting date*termination date*sample period (*F =* 3.80; df = 19, 199; *P* <.0001) (Fig. 8A). The interaction occurred mainly because of differences between early and late termination dates within planting dates 1 and 2 when comparisons were made between sampling periods two and three. For planting date 1, early and late termination date during sampling period two was approaching significance (*F* =3.52; df = 1, 199; *P =* 0.0596), while planting date 1 with a late termination had greater cover crop biomass compared to planting date 1 with an early termination during sampling period three (*F* =48.98; df = 1, 199; *P <*.0001). The same occurred for planting date 2, where no difference between early and late termination date was identified during sampling period two (*F* =0.87; df = 1, 199; *P =* 0.3524), while planting date 2 late had greater cover crop biomass compared to planting date 2 early during sampling period three (*F* =31.60; df = 1, 199; *P <*.0001).

All planting and termination dates treatment combinations for 2020 met the NRCS extended leaf height threshold of 152 mm at the time of the cover crop termination. The extended leaf height varied from 341 mm for planting date 4 with an early cover crop termination date to 589 mm for planting date 1 with a late cover crop termination date. Similar to 2019, the 2020 the cover crop planting date main effect was significant (*F* = 15.76; df = 3, 9; *P =* 0.0006) (Fig. 8B). Planting dates 1 and 2 had greater extended leaf height compared to planting dates 3 and 4, with a numerical decrease in extended leaf height with a delay in cover crop planting date.

Corn grain yields varied from 9,385 kg ha^−1^ (planting date 3) to 11,331 kg ha^−1^ (no-cover crop). Cover crop planting date was the only significant effect (*F =* 3.65; df = 4, 12; *P =* 0.0362). The no-cover crop treatment (11,331 kg ha^−1^) had greater corn yield compared to the planting date 3 (9,385 kg ha^−1^). However, the no-cover crop and planting date 3 were not different from the planting date 1 (10,295 kg ha^−1^), 2 (9,501 kg ha^−1^), and 4 (10,351 kg ha^−1^).

## Discussion

Understanding the impact of cover crop planting and termination date is an important decision-making management strategy to potentially maximize beneficial arthropods and decrease pest pressures in agricultural systems. Our study found that cover crop planting and termination dates significantly impacted the cover crop biomass production. In general, early cover crop planting with a late termination date resulted in greater cover crop biomass accumulation compared to a late cover crop planting with an early termination date. These results are similar to previous studies evaluating biomass with different cover crop management strategies (Mirsky et al. 2017, Webster et al. 2016, Sciarresi et al. 2020, Rosa et al. 2021a). We hypothesized that management strategies that result in greater cover crop biomass would support more arthropod prey resulting in greater arthropod activity. We also hypothesized that treatments with greater cover crop biomass would support more beneficial arthropods that could reduce the pest populations, if pests were present. The results of this two-year field study partially supported our hypotheses with increased Araneae activity in treatments with increased cover crop biomass. NMDS results showed that cover crop biomass significantly impacted arthropod activity in 7 of the 10 sampling periods over the two years of this study. Araneae appeared to be impacted by greater cover crop biomass as a result of the availability of potential prey such as Collembola and Acari. NMDS results showed an increase in Carabidae activity with greater cover crop biomass during sampling periods one and two early in the season. However, the relationship between arthropod taxa and cover crop biomass varied over sampling periods and between years, but no pest pressure was detected in any treatment over the two years of the study. As a result, this study could not evaluate the potential reduction in pest pressure from beneficial arthropods with increasing cover crop biomass.

Manipulating habitat, such as adding a cover crop to a production system to increase habitat diversification, is expected to influence generalist predator mobility, attractiveness, dispersal tendency, alternate food sources, sites for web attachment, and prey accessibility (Symondson 2002). Araneae has been observed to have a strong positive response to increased diversification within fields (Sunderland and Samu 2000, Langellotto and Denno 2004) with the potential to reduce pest populations (Symondson 2002, Michalko and Pekar 2015). Our study findings support this previous literature, where greater Araneae occurred in treatments with more cover crop biomass (cover crop planting date 1 and 2 with late termination) in both years of the study. Araneae is considered one of the most abundant and diversified generalist arthropod predator groups in several cropping systems (Michálek et al. 2017) and the most diversified group of predators in terms of the number of prey species consumed (Pekár and Toft 2015).

Araneae have been reported to catch a wide range of prey (Wise 1993, Oelbermann and Scheu 2009). Most Araneae species prey on other spiders and arthropods, such as Collembola, Aphididae, and Acari (Almeida-Silva 2009, Pekár, 2012, Tsai and Pekár 2019, García et al. 2020). However, due to the changes in prey abundance, the food source of Araneae changes throughout the year (Edgar 1969, Symondson et al. 2002, Saska et al. 2013). In addition, Araneae are known to respond to environmental changes, and some species are highly sensitive to small changes in habitat structure and microclimate factors (Buddle et al. 2000, Marc et al. 1999, Pearce and Venier 2006, Reyes-Maldonado et al. 2018, Maelfait 1996, Honek 1988, 1997). The NMDS results in this study give insights into the impact of cover crop biomass over time on Araneae activity and its relationship with possible prey. Prior to planting corn, the cover crop biomass-Araneae-alternative prey relationship was not clear, with a varying response between years, limiting our ability to draw any conclusions (Figs. 5A–C and 6A–C). However, this relationship changed after corn was planted with cover crop biomass significantly impacting arthropod activity in both years for sampling periods four and five. Of these arthropods, Araneae was positively related to increased cover crop biomass residue during sampling periods four and five in both years (Figs. 5D, E and 6D, E). Araneae increased with Collembola and Acari activity in 2019 during sampling period four. In contrast, Araneae activity in 2020 was not related with Collembola and Acari activity during sampling period four. Increased Collembola and Acari activity co-occurred with an increase in Araneae activity in 2020 during sampling period three. These Araneae-cover crop biomass-possible prey interactions differences between years might be related to the cover crop biomass accumulation differences between years, where a greater cover crop biomass accumulation occurred in 2020 compared to 2019. In addition, Aphididae activity was greater in 2020 during sampling period three which may impact the Araneae-alternative prey interactions. Those findings give insights about a possible cover crop biomass-Araneae-Collembola and Acari interactions, where greater cover crop biomass might be creating a habitat for supporting a prey-predator environment with some changes throughout the growing season.

Cover crops are well-studied as a strategy to reduce weed suppression in cropping systems by physical suppression (Ospitan et al. 2018). However, cover crops can also indirectly contribute to weed management by increasing weed seed predators, such as Carabids. Besides Araneae, NMDS results also show that Carabidae was impacted by the cover crop biomass over time. Despite some differences between years in the impact of cover crop biomass on arthropod activity, Carabidae was positively related to cover crop biomass during sampling periods one and two (Figs. 5 and 6). Similarly to these findings, Shearin et al. (2008) found that treatments with more cover crop biomass (pea/oat with 4,100 to 4,900 kg ha^−1^) supported more *Harpalus rufipes* DeGeer during the cover crop growth period and consequently retained higher Carabidae activity also during the corn growth. Our findings, along with the previous literature (Carmona and Landis 1999, Dunbar et al. 2017, Shearin et al. 2008) suggest that cover crop biomass has an important role in supporting beneficial arthropods with the potential to contribute to ecosystem services for weed suppression and pest management.

In this study, the relationship between the most abundant taxa with cover crop biomass varied over time and between years, potentially as a result of environmental differences between years and sampling periods and the cover crop growth status and arthropod dynamics. Halaj and Wise (2002) reported an increase in carabid beetles, spiders, and Collembola in detritus-addition treatments, but with also variation during the season. When studying cover crop management as cover crop planting and termination dates, it is expected to have treatments with different cover crop biomass accumulation, consequently having different carbon to nitrogen ratios and lignin, which has been reported to dictate the decomposer community (Ruffo and Bollero 2003). The complex interaction between prey-predators-environmental conditions-cover crop growth and decomposition status might be related to the seasonal changes in epigeal arthropods' activity.

Pest pressure was anticipated to decrease as cover crop biomass increased. Our results did not support this hypothesis with pest pressure causing less than 1% injury for all treatments and years of the study. Dunbar et al. (2016) used a non-*Bt* corn hybrid and reported a greater number of true armyworm (*Mythimna unipuncta* (Haworth)) with feeding injury in a cornfield following a rye cover crop compared to no cover crop. The same authors found that common stalk borer [*Papaipema nebris* (Guenee)] was not influenced by the cover crop, and that black cutworm [*Agrotis ipsilon* (Hufnagel)] presence was rare in a cover crop system. Those early-season corn pests are known to feed on cereal rye as cover crop and then have the potential to migrate to corn. The lack of pest pressure in our study might be due to those pests being absent from the system, the presence of beneficial arthropods, and/or the use of *Bt* corn hybrids. The ENREEC Black Light Trap (Knoell 2021) was active from early May through the end of August, and results show that between early May to early July (the timeframe that the corn damage assessments were done in our experiment), true armyworm (2019: 351; 2020: 261) and black cutworm adults (2019: 9; 2020: 6) were present in medium and low populations near the experimental area, respectively (Knoell 2021). In addition, Campos (2021) conducted a four site-year study to evaluate cover crop species and cover crop termination date impact on arthropods in east and central Nebraska with a non-*Bt* corn hybrid used in two site-years of the study and did not find significant pest pressure in any treatment during any site year.

Although arthropod communities are important for an agroecosystem, cash crop yield is the most common metric for evaluating cropping systems’ profitability. As a result, cover crops are often treated as a sustainable tool to be used in the cropping system, but only if the cover crop does not reduce the cash crop yield (Schipanski et al. 2014). Our two-year field study showed that higher cover crop biomass accumulation in treatments with early cover crop planting date and later cover crop termination date reduced corn grain yield compared to treatments with a late cover crop planting date and early cover crop termination date or with no cover crop. Recent studies showed that grass cover crop species utilized before corn could reduce corn yield due to higher nitrogen immobilization by the grass cover crop (Rosa et al. 2021b). Also, greater cover crop biomass might reduce cash crop yield due to greater water use from the cover crop (Rosa et al. 2021a). Utilizing legumes as part of a mixture in a cover crop can modify starter nitrogen rates and water management in a grass cover crop to grass cash crop rotation could be a viable strategy to reduce the potential for negative impacts on yield (Clark et al. 1995, 1997, 2007a,b). Sciarresi et al. (2020) found that in a cover crop-soybean system, the selection of a soybean maturity group could be an option to increase the cover crop growth period for greater biomass accumulation without impacting soybean yield. However, cash crop yield is not the only important profitability parameter that should be evaluated in a cropping system. Ecosystem services, defined as functions provided by an environment that benefit humans (Millennium Ecosystem Assessment 2005) are a strategy to measure the cropping systems’ sustainability and resilience beyond cash crop yield alone (Schipanski et al. 2014). Many ecosystem services are provided when using cover crops that can increase predation levels and reduce insecticide use, all of which may improve the cropping system resilience, yield stability, and reduce external input requirements (Schipanski et al. 2014).

The two-year field study data suggest that integrating an oat and cereal rye grass cover crop that maximizes cover crop biomass production in annual corn rotations presents a potential opportunity to increase beneficial arthropods activity. However, the same strategy can result in a reduction in corn yield. The cover crop biomass-Araneae-alternative preys relationship varied during the growing season and between years. However, early cover crop planting dates combined with late termination dates maximized cover crop biomass accumulation leading to an increase in Carabidae and Araneae activity. Identifying and utilizing management strategies that increase beneficial arthropods can help growers make informed decisions that contribute to beneficial arthropods conservation. Adding cover crops may maintain a balance between predators/prey in an agroecosystem. When a pest is present, predators being conserved due to the use of cover crops may have the potential to naturally decrease the pest pressure. However, increased cover crop biomass was also shown to decrease corn grain yield, indicating that the grass cover crop–corn cash crop system would need a modification in the nitrogen and or water management strategy to prevent potential yield losses.

This is the first study with significant temporal sampling to describe arthropod activity during the cover crop growth and the early corn season as well as its relationship with cover crop biomass. Also, we describe the potential relationship and changing dynamics between Araneae, prey, and cover crop biomass over time. Future cover crop field studies should consider Araneae gut content analysis to determine its potential food sources when influenced by cover crop management strategies that change cover crop biomass. This study also found a decrease in corn yield with an increase of cover crop biomass accumulation. Continued research is needed to evaluate the reduction in corn yield relative to an increase in ecological functions, and the benefits of using cover crops are necessary to understand the trade-off in this system. There is a need to understand the whole system as cover crops add a management dynamic that is not accounted for in a corn-fallow system as well as the variation in environmental conditions and their impact on the cover crop biomass-arthropod activity. Future studies should also consider evaluating a multi-year cover crop system as such practices are likely to alter arthropod activity.

## Supplementary Material

toac090_suppl_Supplementary_Figure_S1

toac090_suppl_Supplementary_Figure_S2

toac090_suppl_Supplementary_Figure_S3

toac090_suppl_Supplementary_Table_S1

toac090_suppl_Supplementary_Table_S2
