# Supplementary figures and images for: Impact of Cover Crop Planting and Termination Dates on Arthropod Activity in the Following Corn
Source: J Econ Entomol. 2022 Jul 4;115(4):1177–90. doi: 10.1093/jee/toac090 (PMC9365511; doi:10.1093/jee/toac090)

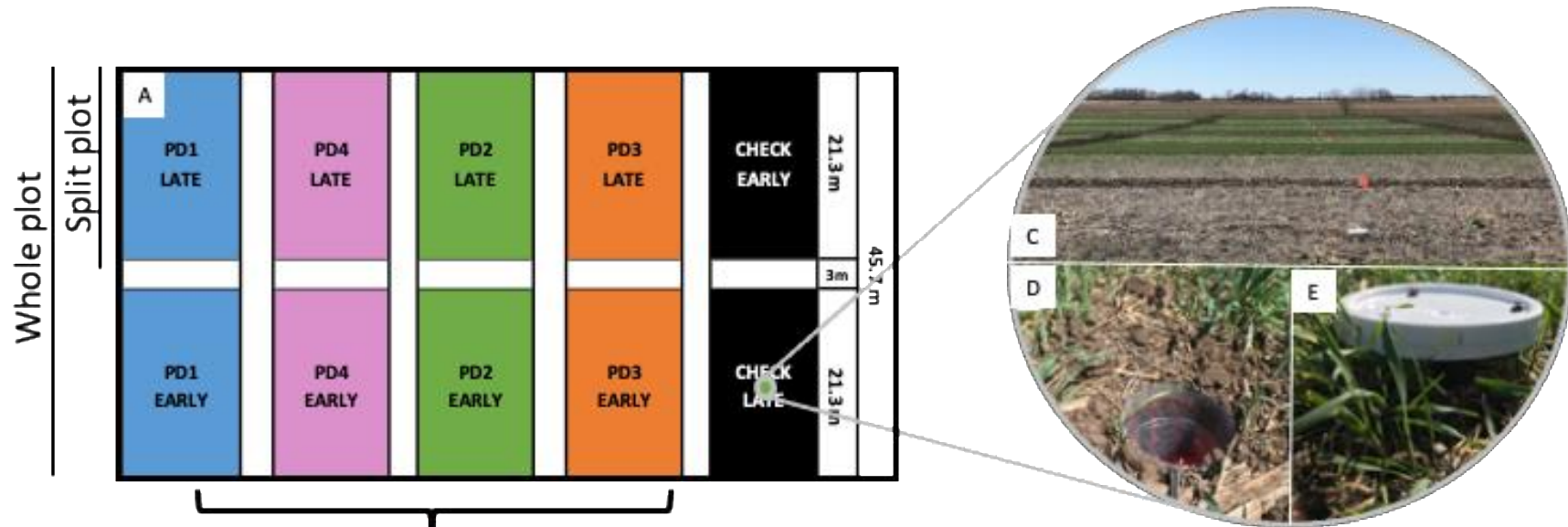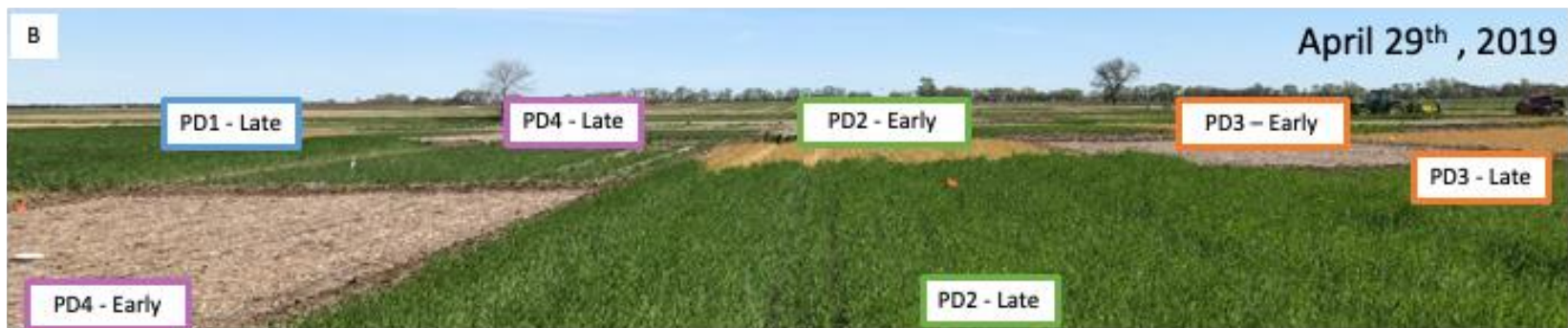

Supplement: toac090_suppl_Supplementary_Figure_S1 [file toac090_suppl_supplementary_figure_s1.pdf]

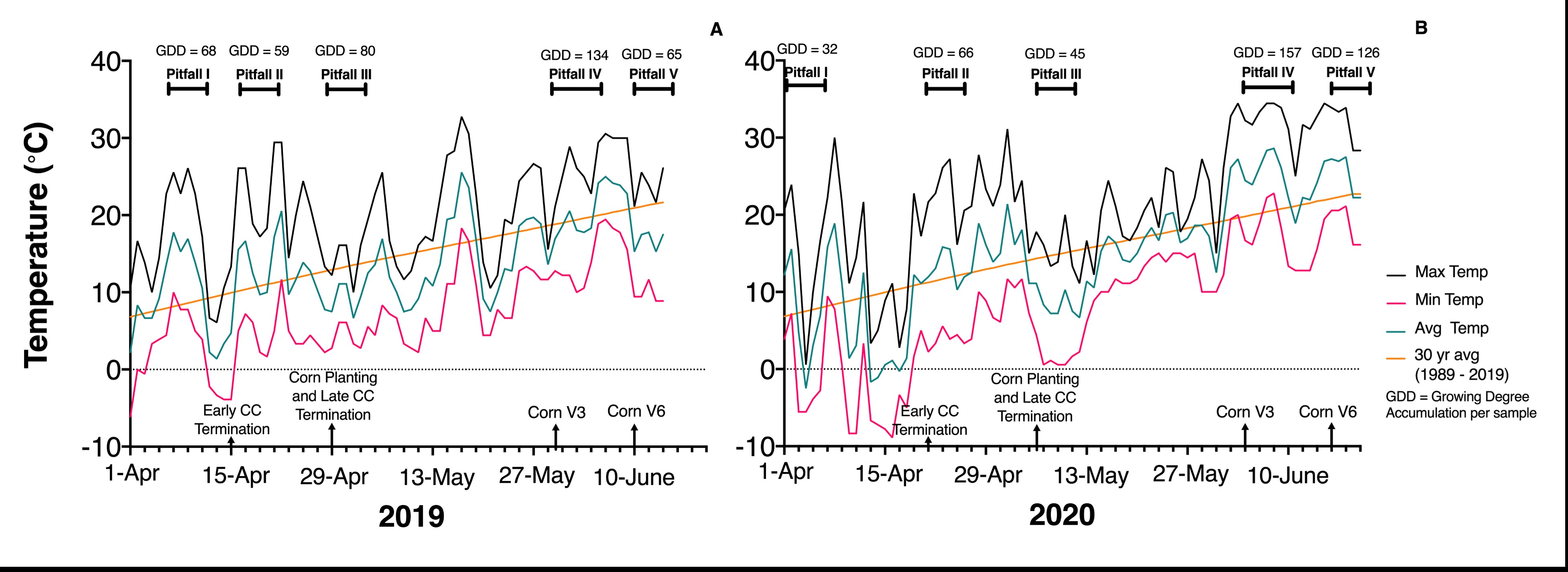

Supplement: toac090_suppl_Supplementary_Figure_S2 [file toac090_suppl_supplementary_figure_s2.jpeg]

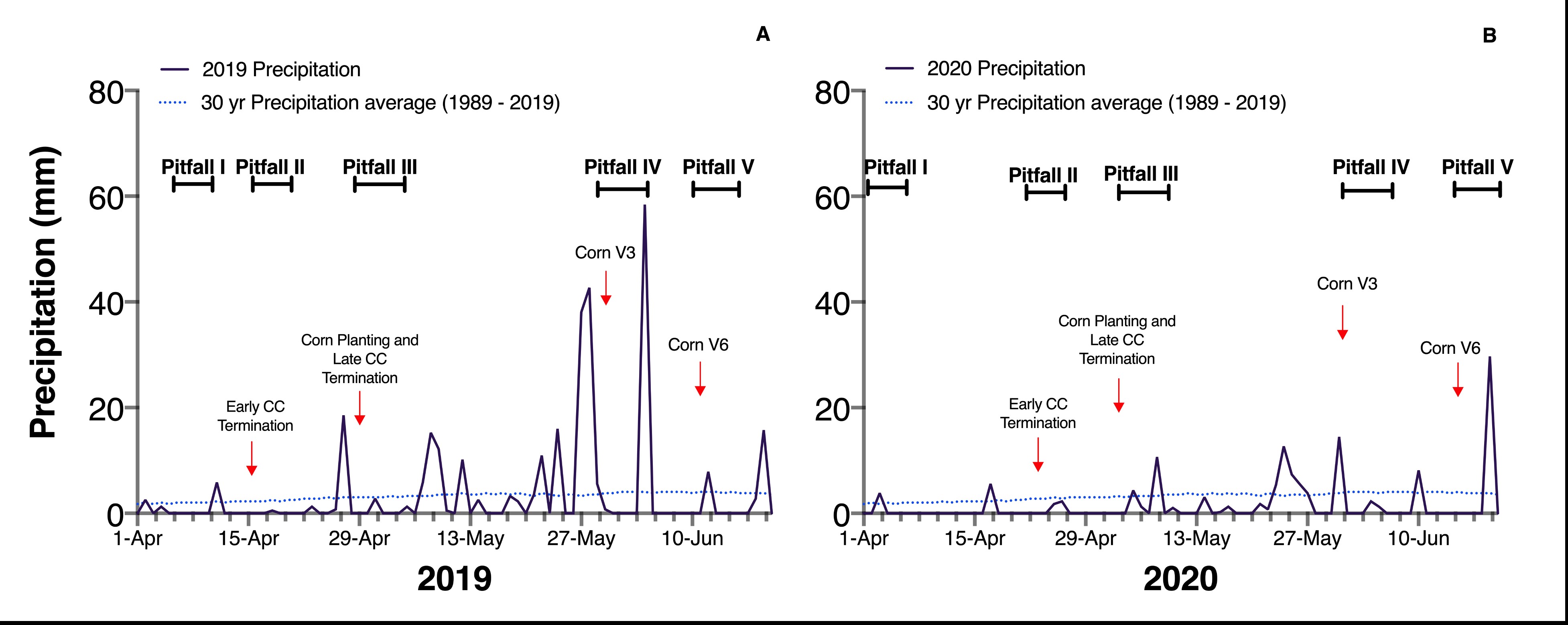

Supplement: toac090_suppl_Supplementary_Figure_S3 [file toac090_suppl_supplementary_figure_s3.jpeg]
