# Supplementary material for: Impact of Cover Crop Planting and Termination Dates on Arthropod Activity in the Following Corn
Source: J Econ Entomol. 2022 Jul 4;115(4):1177–90. doi: 10.1093/jee/toac090 (PMC9365511; doi:10.1093/jee/toac090)
Supplement: toac090_suppl_Supplementary_Table_S1 [file toac090_suppl_supplementary_table_s1.docx]

**Suppl. Table 1.** P-values from generalized linear mixed models used to test the effect of sample, cover crop planting date (PD), cover crop terminating date (TD), year, and its interactions. Significant p-values (<0.05) are shown in bold.

| **Class** | **Insecta** | | | | | | | **Arachnida** | | **Collembola^6^** |
| --- | --- | --- | --- | --- | --- | --- | --- | --- | --- | --- |
| **Order** | **Hemiptera** | **Coleoptera** | | | | **Diptera** | | **Araneae^6^** | **Acari^8^** |  |
| **Family** | **Aphididae^1^** | **Carabidae^2^** | **Staphylinidae^3^** | **Nitidulidae^4^** | **Zopheridae^5^** | **Anthomydae^6^** | **Sciaridae^7^** |  |  |  |
| **Year*PD*TD*Sample** | **-** | - | 0.5353 | 0.7618 | - | 0.9386 | - | 0.7038 | 0.0853 | 0.9369 |
| **PD*TD*Sample** | **-** | 0.8667 | 0.8783 | 0.7331 | 0.5477 | 0.6495 | - | 0.3866 | 0.1987 | 0.5388 |
| **Year*TD*Sample** | **-** | - | 0.9604 | 0.3023 | - | 0.8511 | - | 0.6362 | 0.2652 | 0.4257 |
| **Year*PD*Sample** | **-** | - | 0.2659 | 0.189 | - | 0.8255 | - | 0.8695 | 0.5587 | 0.3797 |
| **Year*PD*TD** | **0.0005***** | - | 0.4131 | 0.1703 | - | 0.8616 | 0.8647 | 0.3665 | 0.4861 | 0.4159 |
| **TD*Sample** | **-** | 0.9904 | 0.3604 | **0.0099***** | 0.3731 | **0.0200**** | - | **0.0011**** | 0.9218 | 0.6645 |
| **PD*Sample** | **-** | 0.9188 | 0.8924 | **0.0033**** | **0.0034**** | 0.219 | - | 0.2115 | **0.0011**** | 0.8789 |
| **Year*Sample** | **-** | - | **0.0185**** | **<.0001** | - | **0.0005***** | - | 0.5770 | **<.0001***** | **<.0001***** |
| **PD*TD** | 0.8711 | 0.9996 | 0.2100 | 0.8408 | 0.0763 | 0.5732 | 0.8486 | 0.3519 | **0.0012**** | 0.2821 |
| **Year*TD** | **<.0001***** | - | **0.0096***** | 0.224 | - | 0.5419 | 0.4954 | 0.7627 | 0.4304 | 0.6240 |
| **Year*PD** | 0.5859 | - | 0.4456 | **0.0243**** | - | 0.3186 | 0.133 | 0.0695 | 0.8658 | **0.0069**** |
| **Sample** | **-** | 0.9843 | **0.0391*** | **<.0001***** | **<.0001***** | **<.0001***** | - | **<.0001***** | **<.0001***** | **<.0001***** |
| **Year** | **<.0001***** | - | **<.0001***** | **0.0001***** | - | 0.0897 | 0.0264 | **<.0001***** | 0.9029 | **<.0001***** |
| **PD** | 0.0018 | 0.4573 | 0.3127 | **0.0121**** | 0.4259 | **0.0027**** | 0.7927 | **<.0001***** | **0.0042**** | 0.5228 |
| **TD** | 0.0043 | 0.9899 | 0.4427 | **0.0126*** | 0.7974 | **0.0039**** | 0.947 | 0.9820 | **0.0010**** | 0.9464 |

^1^Family captured only in pitfall sample 3 (at late cover crop termination)

^2^ Family captured in pitfall samples 4 and 5 (at V3 and V6 corn stage, respectively) during the 2020 growing season.

^3^ Family captured in pitfall samples 4 and 5 (at V3 and V6 corn stage, respectively)

^4^ Family not captured only in pitfall samples 3 (at late cover crop termination)

^5^ Family captured only in the 2020 growing season.

^6^ Family captured in all pitfall samples.

^7^ Family captured only in pitfall samples 4 (at V3 corn stage).

^8^ Family captured only in pitfall samples 4 and 5 (at V3 and V6 corn stage, respectively).

– represents tests not performed due to not enough taxa activity.

* = <.0001 ** = <0.01 *** = <0.05 significant levels.
