# Supplementary material for: Impact of Cover Crop Planting and Termination Dates on Arthropod Activity in the Following Corn
Source: J Econ Entomol. 2022 Jul 4;115(4):1177–90. doi: 10.1093/jee/toac090 (PMC9365511; doi:10.1093/jee/toac090)
Supplement: toac090_suppl_Supplementary_Table_S2 [file toac090_suppl_supplementary_table_s2.docx]

**Suppl. Table 2.** Cover crop dry biomass ($\pm$standard error of the mean) and weed dry biomass ($\pm$standard error of the mean) by sampling periods, cover crop planting date, and cover crop termination date and across 2019 and 2020.

| **Sampling Period** | **Cover Crop Planting Dates** | **Cover Crop Termination Date** | **2019** | | | | | | **2020** | | | | | |
| --- | --- | --- | --- | --- | --- | --- | --- | --- | --- | --- | --- | --- | --- | --- |
|  |  |  | **Cover Crop Dry Biomass (kg ha^-1^) (mean ± SEM)** | | | **Weeds Dry Biomass (kg ha^-1^) (mean ± SEM)** | | | **Cover Crop Dry Biomass (kg ha^-1^) (mean ± SEM)** | | | **Weeds Dry Biomass (kg ha^-1^) (mean ± SEM)** | | |
| One (Before cover crop termination) | NC | Early | 0.0 | ± | 0.0 | 0.0 | ± | 0.0 | 0.0 | ± | 0.0 | 275.8 | ± | 214.1 |
|  | 1 |  | 1438.4 | ± | 522.2 | 0.0 | ± | 0.0 | 719.0 | ± | 245.7 | 149.4 | ± | 100.7 |
|  | 2 |  | 848.1 | ± | 390.1 | 0.0 | ± | 0.0 | 496.3 | ± | 189.8 | 44.4 | ± | 56.7 |
|  | 3 |  | 96.4 | ± | 45.8 | 0.0 | ± | 0.0 | 107.4 | ± | 111.8 | 35.0 | ± | 49.2 |
|  | 4 |  | 78.9 | ± | 55.4 | 4.5 | ± | 11.0 | 121.2 | ± | 58.5 | 30.3 | ± | 47.3 |
|  | NC | Late | 0.0 | ± | 0.0 | 0.0 | ± | 0.0 | 0.0 | ± | 0.0 | 174.9 | ± | 241.9 |
|  | 1 |  | 1129.8 | ± | 302.2 | 0.0 | ± | 0.0 | 697.4 | ± | 233.8 | 145.3 | ± | 147.3 |
|  | 2 |  | 754.8 | ± | 307.0 | 0.0 | ± | 0.0 | 411.1 | ± | 68.0 | 133.2 | ± | 93.5 |
|  | 3 |  | 92.4 | ± | 38.6 | 0.0 | ± | 0.0 | 146.4 | ± | 97.2 | 110.3 | ± | 78.4 |
|  | 4 |  | 64.6 | ± | 45.3 | 0.0 | ± | 0.0 | 114.0 | ± | 42.1 | 47.1 | ± | 59.5 |
| Two (At early cover crop termination) | NC | Early | 0.0 | ± | 0.0 | 0.0 | ± | 0.0 | 0.0 | ± | 0.0 | 257.7 | ± | 269.0 |
|  | 1 |  | 2044.3 | ± | 717.4 | 0.0 | ± | 0.0 | 2623.8 | ± | 640.6 | 39.0 | ± | 45.3 |
|  | 2 |  | 851.7 | ± | 211.3 | 0.0 | ± | 0.0 | 1661.1 | ± | 489.9 | 80.1 | ± | 102.8 |
|  | 3 |  | 109.0 | ± | 41.3 | 0.0 | ± | 0.0 | 512.0 | ± | 278.6 | 105.0 | ± | 140.1 |
|  | 4 |  | 63.7 | ± | 21.4 | 0.0 | ± | 0.0 | 515.3 | ± | 264.0 | 18.8 | ± | 37.7 |
|  | NC | Late | 0.0 | ± | 0.0 | 0.0 | ± | 0.0 | 0.0 | ± | 0.0 | 198.5 | ± | 148.0 |
|  | 1 |  | 1786.0 | ± | 263.0 | 0.0 | ± | 0.0 | 2038.5 | ± | 796.7 | 55.2 | ± | 110.3 |
|  | 2 |  | 975.5 | ± | 288.7 | 0.0 | ± | 0.0 | 1948.3 | ± | 407.6 | 23.5 | ± | 27.2 |
|  | 3 |  | 124.2 | ± | 34.5 | 0.0 | ± | 0.0 | 721.2 | ± | 585.7 | 79.4 | ± | 75.4 |
|  | 4 |  | 56.1 | ± | 21.7 | 0.0 | ± | 0.0 | 453.4 | ± | 189.0 | 1.3 | ± | 2.7 |

| **Sampling Period** | **Cover Crop Planting Dates (PD)** | **Cover Crop Termination Date (TD)** | **2019** | | | | | | **2020** | | | | | |
| --- | --- | --- | --- | --- | --- | --- | --- | --- | --- | --- | --- | --- | --- | --- |
|  |  |  | **Cover Crop Dry Biomass (kg ha^-1^) (mean ± SEM)** | | | **Weeds Dry Biomass (kg ha^-1^) (mean ± SEM)** | | | **Cover Crop Dry Biomass (kg ha^-1^) (mean ± SEM)** | | | **Weeds Dry Biomass (kg ha^-1^) (mean ± SEM)** | | |
| Three (At late cover crop termination) | NC | Early | 0.0 | ± | 0.0 | 15.9 | ± | 39.0 | 0.0 | ± | 0.0 | 53.8 | ± | 78.7 |
|  | 1 |  | 1273.8 | ± | 328.5 | 0.0 | ± | 0.0 | 1720.3 | ± | 524.7 | 0.0 | ± | 0.0 |
|  | 2 |  | 796.1 | ± | 228.6 | 0.0 | ± | 0.0 | 932.5 | ± | 391.5 | 0.0 | ± | 0.0 |
|  | 3 |  | 142.6 | ± | 132.5 | 0.0 | ± | 0.0 | 346.5 | ± | 251.3 | 0.0 | ± | 0.0 |
|  | 4 |  | 50.7 | ± | 25.6 | 0.0 | ± | 0.0 | 318.2 | ± | 195.7 | 0.0 | ± | 0.0 |
|  | NC | Late | 0.0 | ± | 0.0 | 21.1 | ± | 22.6 | 0.0 | ± | 0.0 | 43.1 | ± | 51.4 |
|  | 1 |  | 3365.2 | ± | 717.2 | 0.0 | ± | 0.0 | 3873.8 | ± | 912.8 | 35.0 | ± | 41.3 |
|  | 2 |  | 2741.3 | ± | 847.3 | 0.0 | ± | 0.0 | 2662.1 | ± | 1099.6 | 0.0 | ± | 0.0 |
|  | 3 |  | 493.8 | ± | 187.7 | 4.5 | ± | 11.0 | 1746.5 | ± | 1092.4 | 0.0 | ± | 0.0 |
|  | 4 |  | 356.6 | ± | 90.9 | 2.0 | ± | 4.9 | 1081.8 | ± | 593.2 | 0.0 | ± | 0.0 |
| Four (At V3 corn stage) | NC | Early | 0.0 | ± | 0.0 | 0.0 | ± | 0.0 | 0.0 | ± | 0.0 | 14.8 | ± | 29.6 |
|  | 1 |  | 887.6 | ± | 353.3 | 0.0 | ± | 0.0 | 1209.0 | ± | 331.5 | 0.0 | ± | 0.0 |
|  | 2 |  | 666.5 | ± | 414.6 | 0.0 | ± | 0.0 | 536.2 | ± | 129.1 | 0.0 | ± | 0.0 |
|  | 3 |  | 4.9 | ± | 12.1 | 0.0 | ± | 0.0 | 179.0 | ± | 233.6 | 0.0 | ± | 0.0 |
|  | 4 |  | 4.9 | ± | 12.1 | 0.0 | ± | 0.0 | 81.4 | ± | 109.4 | 0.0 | ± | 0.0 |
|  | NC | Late | 0.0 | ± | 0.0 | 0.2 | ± | 0.4 | 0.0 | ± | 0.0 | 16.1 | ± | 32.3 |
|  | 1 |  | 2362.3 | ± | 583.5 | 7.0 | ± | 17.2 | 3550.9 | ± | 682.3 | 8.1 | ± | 16.1 |
|  | 2 |  | 2228.7 | ± | 929.2 | 0.0 | ± | 0.0 | 2009.6 | ± | 403.8 | 0.0 | ± | 0.0 |
|  | 3 |  | 390.7 | ± | 125.8 | 0.1 | ± | 0.2 | 1373.1 | ± | 1009.1 | 0.0 | ± | 0.0 |
|  | 4 |  | 162.8 | ± | 72.4 | 0.0 | ± | 0.0 | 984.9 | ± | 655.8 | 0.0 | ± | 0.0 |

| **Sampling Period** | **Cover Crop Planting Dates (PD)** | **Cover Crop Termination Date (TD)** | **2019** | | | | | | **2020** | | | | | |
| --- | --- | --- | --- | --- | --- | --- | --- | --- | --- | --- | --- | --- | --- | --- |
|  |  |  | **Cover Crop Dry Biomass (kg ha^-1^) (mean ± SEM)** | | | **Weeds Dry Biomass (kg ha^-1^) (mean ± SEM)** | | | **Cover Crop Dry Biomass (kg ha^-1^) (mean ± SEM)** | | | **Weeds Dry Biomass (kg ha^-1^) (mean ± SEM)** | | |
| Five (At V6 corn stage) | NC | Early | 0.0 | ± | 0.0 | 0.0 | ± | 0.0 | 0.0 | ± | 0.0 | 157.4 | ± | 276.8 |
|  | 1 |  | 345.4 | ± | 191.5 | 0.0 | ± | 0.0 | 705.7 | ± | 343.1 | 150.7 | ± | 301.4 |
|  | 2 |  | 163.7 | ± | 401.0 | 0.0 | ± | 0.0 | 283.2 | ± | 94.8 | 0.0 | ± | 0.0 |
|  | 3 |  | 0.0 | ± | 0.0 | 0.0 | ± | 0.0 | 43.7 | ± | 36.3 | 39.0 | ± | 78.0 |
|  | 4 |  | 0.0 | ± | 0.0 | 0.0 | ± | 0.0 | 12.1 | ± | 24.2 | 55.8 | ± | 111.7 |
|  | NC | Late | 0.0 | ± | 0.0 | 0.0 | ± | 0.0 | 18.8 | ± | 37.7 | 72.7 | ± | 145.3 |
|  | 1 |  | 2589.7 | ± | 899.8 | 0.0 | ± | 0.0 | 3106.4 | ± | 1013.7 | 21.5 | ± | 43.1 |
|  | 2 |  | 1837.6 | ± | 654.2 | 0.0 | ± | 0.0 | 2103.7 | ± | 752.6 | 0.0 | ± | 0.0 |
|  | 3 |  | 186.6 | ± | 184.1 | 0.0 | ± | 0.0 | 1252.7 | ± | 844.7 | 309.5 | ± | 618.9 |
|  | 4 |  | 0.0 | ± | 0.0 | 0.0 | ± | 0.0 | 898.1 | ± | 260.6 | 0.0 | ± | 0.0 |

NC represents the no-cover crop treatment (check).
